# Inhibiting Mitochondrial Cytochrome *c* Oxidase Downregulates Gene Transcription After Traumatic Brain Injury in *Drosophila*

**DOI:** 10.3389/fphys.2021.628777

**Published:** 2021-03-15

**Authors:** Ekta J. Shah, Maik Hüttemann, Thomas H. Sanderson, Katherine Gurdziel, Douglas M. Ruden

**Affiliations:** ^1^Department of Pharmacology, Wayne State University School of Medicine, Detroit, MI, United States; ^2^Center for Molecular Medicine and Genetics, Wayne State University School of Medicine, Detroit, MI, United States; ^3^Department of Emergency Medicine, University of Michigan Medical School, Ann Arbor, MI, United States; ^4^Office of the Vice President of Research, Wayne State University, Detroit, MI, United States; ^5^Institute of Environmental Health Sciences, Wayne State University, Detroit, MI, United States; ^6^Department of Obstetrics and Gynecology, Wayne State University, Detroit, MI, United States

**Keywords:** traumatic brain injury, sex-differences, gene expression, near-infrared light, mitochondria

## Abstract

Traumatic brain injuries (TBIs) caused by a sudden impact to the head alter behavior and impair physical and cognitive function. Besides the severity, type and area of the brain affected, the outcome of TBI is also influenced by the patient’s biological sex. Previous studies reporting mitochondrial dysfunction mainly focused on exponential reactive oxygen species (ROS) generation, increased mitochondrial membrane potential, and altered mitochondrial dynamics as a key player in the outcome to brain injury. In this study, we evaluated the effect of a near-infrared (NIR) light exposure on gene expression in a *Drosophila* TBI model. NIR interacts with cytochrome *c* oxidase (COX) of the electron transport chain to reduce mitochondrial membrane potential hyperpolarization, attenuate ROS generation, and apoptosis. We subjected *w*^1118^ male and female flies to TBI using a high-impact trauma (HIT) device and subsequently exposed the isolated fly brains to a COX-inhibitory wavelength of 750 nm for 2 hours (hr). Genome-wide 3′-mRNA-sequencing of fly brains revealed that injured *w*^1118^ females exhibit greater changes in transcription compared to males at 1, 2, and 4 hours (hr) after TBI. Inhibiting COX by exposure to NIR downregulates gene expression in injured females but has minimal effect in injured males. Our results suggest that mitochondrial COX modulation with NIR alters gene expression in *Drosophila* following TBI and the response to injury and NIR exposure varies by biological sex.

## Introduction

Traumatic brain injury (TBI) results from a violent blow or jolt to the head causing a wide range of physical and psychological effects (Finnie and Blumbergs, 2002; Ciuffreda et al., 2016; Dale Horne, 2018). There are estimated to be about 2.5 million TBI cases every year that require emergency department visits or result in death (Taylor et al., 2017). The most common causes of TBI include sports, motor vehicle accidents, falls, and violence (Hiebert et al., 2015) with the symptoms of the injury depending on the type and area of the brain affected (Morries et al., 2015). TBI is a heterogenous disorder consisting of primary damage resulting from direct mechanical forces followed by secondary damage centered on mitochondrial dysfunction leading to neuronal death (Cheng et al., 2012).

Mitochondria are membrane-bound organelles distributed throughout the brain cytosol and responsible for energy production using the electron transport chain (ETC) (Kim et al., 2017). The ETC is a series of protein complexes made of NADH dehydrogenase (complex I), succinate dehydrogenase (complex II), ubiquinone, *bc*_1_-complex (complex III), cytochrome *c* (Cyt*c*), and cytochrome *c* oxidase (COX; complex IV) (Hüttemann et al., 2008). Complexes I, III, and IV pump protons across the inner mitochondrial membrane to generate the mitochondrial membrane potential (ΔΨ_m_) (Hüttemann et al., 2008) which is utilized by ATP synthase (complex V) to synthesize ATP from ADP and phosphate (Hüttemann et al., 2008; Sanderson et al., 2013). An optimal physiological ΔΨ_m_ between 120–140 mV allows efficient ATP production and minimal reactive oxygen species (ROS) generation from complexes I and III (Sanderson et al., 2013). Disruption of ΔΨ_m_ is considered as an indicator of mitochondrial damage causing decreased respiration, decreased ATP production, increased ROS generation and induction of apoptosis by efflux of macromolecules like Cyt*c* and caspase-9/caspase-3 cascade activation (Singh et al., 2006; Watts, 2016).

Mitochondrial impairment has been shown to play a key role in several neurodegenerative disorders including Alzheimer’s disease (AD), Parkinson’s disease (PD), amyotrophic lateral sclerosis (ALS), ischemic brain injury, and stroke (Reddy, 2009; Sanderson et al., 2013, 2018; Strubakos et al., 2020). Following TBI using a controlled cortical impact model, mitochondrial dysfunction and calcium perturbation was observed in male rats (Xiong et al., 1997). A significant decrease in mitochondrial oxidative phosphorylation and calcium buffering capacity was observed in male mice 3 hr post-TBI (Singh et al., 2006). These mice also show structural damage to isolated mitochondria and an increase in oxidative stress (Singh et al., 2006). A *Drosophila* model of TBI also showed significant decrease in ATP production which was observed 24 hr after injury (Sen et al., 2017). It is evident that production of ROS, hyperpolarization of ΔΨ_m_ beyond the physiological range, and caspase activation induce mitochondrial damage following TBI (Lifshitz et al., 2004; Kim et al., 2017). Additionally, biological sex has been shown to influence mitochondrial function (Demarest and McCarthy, 2015; Ventura-Clapier et al., 2017) and the outcome to TBI (Leitgeb et al., 2011; Gupte et al., 2019) but the role of sex-differences in mitochondrial function in response to TBI has not been studied. In a previous study, we observed sex differences in mitochondrial gene transcription and oxidation in *Drosophila* subjected to trauma using the high-impact trauma device (HIT-device) (Katzenberger et al., 2015; Shah et al., 2020). Thus, efforts to attenuate mitochondrial damage have been increasingly studied in TBI, as mitochondrial maintenance could possibly preserve brain function (Watts, 2016). However, most pharmacological approaches to circumvent mitochondrial damage suffer from a critical issue: the ability of drugs to cross the blood-brain barrier and attain effective concentrations in the injured tissue (Sanderson et al., 2013).

In the current study, we explore a non-pharmacological approach of preserving mitochondrial damage in TBI using near-infrared (NIR) light. Photobiomodulation (PBM) or the use of NIR has been studied as a therapeutic alternative in animal and human TBI to protect tissue from dying, increase mitochondrial function, improve blood flow, stimulate healing and tissue oxygenation (Naeser et al., 2011; Hamblin, 2018). PBM involves shining red or NIR light onto the head where the light penetrates into the brain and is absorbed by specific chromophores (Hamblin, 2018). NIR has been shown to interact with cytochrome *c* oxidase (COX), the terminal complex of the ETC (Sanderson et al., 2018; Strubakos et al., 2020). COX contains several chromophores including two copper centers that act as photoacceptors for NIR and absorb light in the range of 700–1000 nm (Karu and Afanas’eva, 1995). Many studies in animal models of TBI using NIR from light-emitting diodes (LEDs) have found improved neurological and cognitive function and reduced inflammation and cell death in brain post-exposure (Naeser et al., 2011; Hamblin, 2018). *Drosophila* exposed to 670 nm NIR showed increased ATP production and reduced inflammation with age (Begum et al., 2015) whereas irradiating *Drosophila pink1* mutants at 808 nm rescued mitochondrial defects (Vos et al., 2013). Recent studies in animal models of stroke and ischemia/reperfusion injury have found that NIR exposure at 750 nm inhibits COX activity (Sanderson et al., 2018), reduces mitochondrial respiratory and ΔΨ_m_, and prevents ROS generation (Sanderson et al., 2018; Strubakos et al., 2020). In line with this evidence, we sought to investigate the effect of modulating mitochondrial COX activity with 750 nm NIR exposure in both sexes of a *Drosophila* model of mild traumatic brain injury (mTBI). *Drosophila* brain is enclosed in an exoskeleton consisting of chitin which strongly absorbs NIR and although previous studies found other wavelengths to penetrate fly and mammalian tissues effectively, we found 750 nm NIR penetration through the exoskeleton ineffective. Thus, we isolated fly brains from *w*^1118^ male and female flies inflicted with trauma using the HIT-device at control, 1, 2, and 4 hr post-TBI and exposed them to NIR at 750 nm for 2 hr. Post-treatment, we assessed change in gene expression separated by sex and found an overall downregulation in transcription in *w*^1118^ females in response to NIR exposure. As compared to *w*^1118^ females, we saw fewer changes in *w*^1118^ males in response to TBI and NIR exposure. These data suggest that outcome to TBI differs between sexes in *Drosophila* exposed to NIR, which could be a result of sex-differences in mitochondrial function. This is the first study to investigate the response of mitochondrial COX inhibition using NIR light at a single wavelength in both sexes post-TBI within 4 hr.

## Materials and Methods

### Fly Stocks and Crosses

*w*^1118^ stock were obtained from the Bloomington *Drosophila* Stock Center and *repo*-GFP stock was a gift from Dr. Laura Buttitta (University of Michigan). Fly stocks were stored at 25°C at constant humidity and fed with standard sugar/yeast/agar medium. All assays were performed on adult mated flies (10–14 days old).

### Traumatic Brain Injury

Both sexes of *w*^1118^ and *repo*-GFP flies were subjected to a single strike full body trauma using a modified HIT device with the impact arm constrained to a 45° angle (Katzenberger et al., 2013; Sen et al., 2017). This device inflicts mild TBI to the flies. No more than 50 flies were placed in a plastic vial before being confined to the bottom quarter of the vial by a stationary cotton ball. Upon deflection and release of the spring, the vial rapidly contacts a styrofoam pad delivering a mechanical force to the flies as they contact the vial wall and rebound causing closed head trauma.

### NIR Light Emitting Diodes and Exposure

Diodes (epoxy lens type infrared illuminator LED750-66-60, Roithner Lasertechnik, Vienna, Austria) were mounted on heat sinks (black aluminum, 47 × 20 for LED array 60 chips) together with a small fan (EC3010M05X; Evercool, New Taipei City, Taiwan) operated in reverse mode to avoid any heating of the brain tissue (Sanderson et al., 2018). The diodes were operated with an energy density of 100 mW/cm^2^. Brains were dissected from control flies or flies exposed to trauma 1, 2, and 4 hr after injury. Approximately 10–15 fly brains were dissected for each condition and placed in a 35 mm petri dish containing cold Schneider’s media. The petri dish was then placed under the 750 nm diode within 2 cm distance and exposed to NIR for 2 hr. Post-exposure, brains were immediately processed for either RNA collection or confocal imaging.

### RNA Isolation

Total RNA was extracted from *w*^1118^ single fly brains using QIAzol^®^ lysis reagent and Direct-zol^TM^ RNA MicroPrep kit (Zymo Research) following manufacturer’s instructions.

### 3′-mRNA Expression Analysis

Expression analysis was conducted in collaboration with the Wayne State University Genome Sciences Core. Three biological replicates were used for each condition (Shah et al., 2020).

QuantSeq 3′-mRNA-Seq Library Prep Kit FWD for Illumina (Lexogen) was used to generate libraries of sequences close to the 3′ end of polyadenylated RNA from 15 ng of total RNA isolated from single fly brain in 5 μl of nuclease-free water following the low-input protocol. Library aliquots were assessed for overall quality using the ScreenTape for the Agilent 2200 TapeStation and quantified using Qubit^TM^ 1X dsDNA HS Assay kit (Invitrogen). Barcoded libraries were normalized to 2 nM before sequencing at 300 pM on one lane of a NovaSeq 6000 SP flow cell. After de-multiplexing with Illumina’s CASAVA 1.8.2 software, the 50 bp reads were aligned to the *Drosophila* genome (Build dm3) with STAR_2.4 (Dobin et al., 2013) and tabulated for each gene region (Anders et al., 2015). Differential gene expression analysis was used to compare transcriptome changes between conditions using edgeR v.3.22.3 (Robinson et al., 2010) and transcripts were defined as significantly differentially expressed at absolute log2 fold change (| log2 FC|) > 1 with an false discovery rate (FDR) < 0.05. Significant gene expression changes were submitted for gene ontology (GO) analyses using RDAVID (Fresno and Fernandez, 2013) for the following categories: GOTERM_BP_ALL, GOTERM_MF_ALL, UP_KEYWORDS, GOTERM_BP_DIRECT, and GOTERM_MF_DIRECT.

### Heatmaps

Heatmaps were generated using Java Treeview (Saldanha, 2004). Counts representing the number of reads mapped to each gene were obtained using HTSeq (Anders et al., 2015) from STAR alignments (Dobin et al., 2013) before normalization. To normalize, a scaling factor was determined by dividing the uniquely mapped reads for each sample by the sample with the highest uniquely mapped number of reads. The scaling factor was multiplied to each gene count for the sample. The log2 of the normalized averaged counts for all three replicates is represented for each condition on the orange scale (0–10). The log2 fold change, represented on yellow-blue scale (0–6), for each gene is obtained from differential expression analysis across all three replicates (Robinson et al., 2010). Genes significant (| log2 FC| > 1, *p*-value < 0.05) in at least one time point are indicated in black text.

### Confocal Imaging

Between 10–15 adult repo-GFP flies for each sex (control and every time point post-TBI) were inflicted with TBI using the HIT-device (Katzenberger et al., 2013; Shah et al., 2020). Flies were anesthetized with CO_2_ before brains were dissected in 1×PBS (phosphate-buffered saline) and fixed for 5 min with 4% PFA (paraformaldehyde). Fixed brains were mounted using Prolong gold antifade mounting media to visualize changes in GFP expression using a confocal microscope (Zeiss LSM 800) at the Microscopy, Imaging and Cytometry Resources Core at Wayne State University, School of Medicine. Average fluorescence intensity for all brains in each condition was calculated using ImageJ (Schneider et al., 2012) in a blinded study. All data are represented as means ± SEM. Statistical analyses (One-way ANOVA with Dunnett’s multiple comparisons test) were performed using GraphPad Prism to compute statistical significance (*p* < 0.05) between groups.

## Results

### Gene Transcription in TBI Inflicted Flies Is Downregulated After Exposure to NIR

**FIGURE 1:**
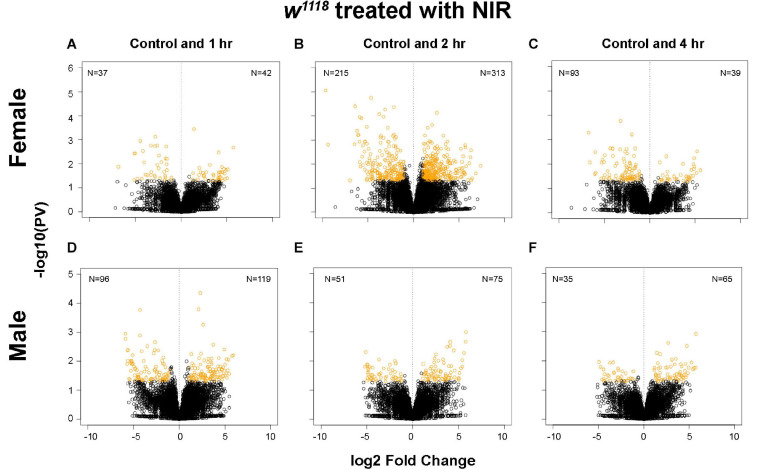
Plot depicting transcriptional changes after NIR exposure in *w*^1118^ injured male and female flies. Volcano plots depicting log2 fold change and –log10(PV:*p*-value) of differentially expressed genes at 1, 2, and 4 hr after injury and subsequent NIR exposure compared to control for females **(A–C)** and males **(D–F)**. The number of significantly upregulated and downregulated gene changes are indicated in each plot (| log2FC| > 1; *p*-value < 0.05). Injured *w*^1118^ females show more transcriptional changes in response to NIR exposure than males.

Several studies in experimental models of brain injury have shown structural and functional damage to mitochondria being an early event in TBI which leads to activation of cell death pathways (Fischer et al., 2016). In a previous study, we demonstrated an upregulation in gene transcription involved with immune response, cytoskeleton organization and apoptosis after injury in *Drosophila* (Shah et al., 2020). Moreover, we have also shown sex-differences in mitochondrial stress and gene transcription in response to TBI (Shah et al., 2020). To identify gene expression changes after attenuation of mitochondria response by COX-inhibitory NIR exposure, we generated 3′-mRNA-Seq libraries from isolated *w*^1118^ male and female fly brains at control and 1-, 2-, and 4-hr post-injury (single strike) time points. Differential gene expression analysis shows significant changes in both sexes after TBI and NIR exposure (Figure 1) with females (Figures 1A–C) exhibiting more transcriptional changes than males (Figures 1D–F). Gene expression changes in response to TBI were less pronounced in both sexes exposed to NIR as compared to flies not treated with NIR (Shah et al., 2020) at all 3 time-points (| log FC| > 2; *p*-value < 0.05).

**FIGURE 2:**
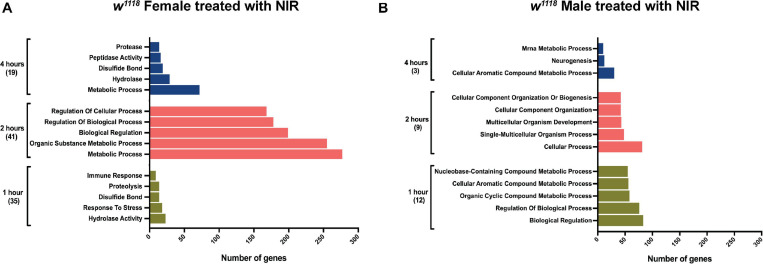
Gene ontology (GO) enrichment in *w*^1118^ injured flies exposed to NIR. Significant genes (| log2FC| > 1; *p*-value < 0.05) identified from sequencing were classified for their biological functions using RDAVID. The plot shows top five biological processes enriched in injured females **(A)** and males **(B)** after NIR exposure at each time-point. The total number of GO terms differentially regulated at each time-point is indicated in parenthesis.

Significant genes identified from 3′-mRNA-Seq were classified for their biological functions using RDAVID (Huang et al., 2009; Fresno and Fernandez, 2013) and several GO categories were found to be changed in both sexes exposed to NIR in response to TBI (Figure 2 and Tables 1, 2). A subset of GO categories significantly altered in both sexes are indicated in Figure 2 wherein it is observed that females have more processes affected than males at all three time-points. In *w*^1118^ females exposed to NIR, the highest number of significant categories (FDR < 0.05) were altered 2 hr after injury (41 GO terms) (Figure 2A) and we also observed significant changes in GO terms for “Humoral immune response,” “Defense response,” “Response to stress,” and “Detection of light stimulus” (Figure 2A, Table 1, and Supplementary Data 1). In our previous study, we observed GO processes including “Immune response,” “Mitochondrial organization,” and “Programmed cell death” significantly altered after TBI in *w*^1118^ females untreated with NIR (Shah et al., 2020). Here, we found no change in any of these processes in females treated with NIR after TBI indicating a positive effect of the COX-inhibitory NIR exposure that could help minimize aberrant gene transcription after brain injury. However, for *w*^1118^ males exposed to NIR, there were fewer significant changes observed than for females, and gene expression related to “nervous system development” and “neurogenesis” was altered after NIR exposure (Figure 2B, Table 2, and Supplementary Data 1). Previous studies involving the use of PBM for TBI patients have also reported downregulation of immune response and upregulation of neurogenesis as an effect of NIR treatment (Hennessy and Hamblin, 2017; Santos et al., 2018). There was no overlap in biological processes affected across all three TBI time-points shared by both sexes. Additionally, we also exposed control fly brains to NIR for both sexes and found that female controls had more significant GO enrichment (18 categories) as compared to male controls (7 categories) (Supplementary Data 1). For female controls, GO terms like “Neurogenesis,” “Oxidation-Reduction Process,” and “Intracellular Transport” were altered whereas for male controls “Nuclear Transport” and “Nucleic Acid Metabolic Process” were changed in response to NIR exposure. Neither sexes showed alteration in immune response or mitochondrial organization (Supplementary Data 1).

**TABLE 1:** Gene ontology terms significantly (FDR < 0.05) changed in response to traumatic brain injury in *w*^1118^ female flies treated with NIR.

Rank	GOBPID	Term	Fold enrichment	FDR
**A: Selected GO terms differentially regulated in *w*^1118^ females treated with NIR after 1 hr of injury**				

1	UP_KEYWORDS	Antibiotic	66.29	<0.01
2	UP_KEYWORDS	Secreted	7.11	<0.01
3	UP_KEYWORDS	Antimicrobial	38.67	<0.01
4	GO:0019731	Antibacterial humoral response	36.78	<0.01
5	GO:0006959	Humoral immune response	35.24	<0.01
17	UP_KEYWORDS	Polymorphism	9.28	<0.01
18	GO:0006508	Proteolysis	3.92	<0.01
30	GO:0009617	Response to bacterium	14.71	0.0289
32	GO:0045087	Innate immune response	8.13	0.0374
33	GO:0006952	Defense response	3.71	0.0380

**B: Selected GO terms differentially regulated in *w*^1118^ females treated with NIR after 2 h of injury**				

1	GO:0048583	Regulation of response to stimulus	1.76	<0.01
2	GO:0010646	Regulation of cell communication	1.78	<0.01
3	GO:0023051	Regulation of signaling	1.78	<0.01
4	UP_KEYWORDS	Vision	5.32	<0.01
5	GO:0009966	Regulation of signal transduction	1.84	<0.01
19	GO:0009583	Detection of light stimulus	4.01	<0.01
21	GO:0071482	Cellular response to light stimulus	4.92	<0.01
27	GO:0050794	Regulation of cellular process	1.23	0.0158
28	GO:0016056	Rhodopsin mediated signaling pathway	7.50	0.0168
32	GO:0050789	Regulation of biological process	1.21	0.0196
37	GO:0030162	Regulation of proteolysis	2.82	0.0376

**C: Selected GO terms differentially regulated in *w*^1118^ females treated with NIR after 4 h of injury**				

1	UP_KEYWORDS	Protease	4.31	<0.01
2	UP_KEYWORDS	Disulfide bond	3.16	<0.01
3	UP_KEYWORDS	Hydrolase	2.04	<0.01
5	GO:0006508	Proteolysis	3.04	0.0133
10	GO:0006040	Amino sugar metabolic process	5.63	0.0230
13	GO:0008152	Metabolic process	1.26	0.0273
17	GO:0004252	Serine-type endopeptidase activity	3.25	0.0447
18	GO:0006022	Aminoglycan metabolic process	4.89	0.0464

*GO terms were sorted based on FDR and ranked accordingly. Tables show selected GO terms changed in females after injury. GOBPID is the ID of the biological process in GO database.*

**TABLE 2:** Gene ontology terms significantly (FDR < 0.05) changed in response to traumatic brain injury in *w*^1118^ male flies treated with NIR.

Rank	GOBPID	Term	Fold enrichment	FDR
**A: Selected GO terms differentially regulated in *w*^1118^ males treated with NIR after 1 h of injury**				

1	GO:0090304	Nucleic acid metabolic process	1.65	<0.01
2	GO:0022613	Ribonucleoprotein complex biogenesis	3.34	<0.01
3	GO:0016070	Rna metabolic process	1.66	<0.01
4	GO:0006139	Nucleobase-containing compound metabolic process	1.50	0.0171
5	GO:1901360	Organic cyclic compound metabolic process	1.47	0.0182
6	GO:0065007	Biological regulation	1.31	0.0244
7	GO:0006950	Response to stress	1.74	0.0301
8	GO:0046483	Heterocycle metabolic process	1.46	0.0325
9	GO:0006725	Cellular aromatic compound metabolic process	1.45	0.0339
10	GO:0050789	Regulation of biological process	1.33	0.0344
11	GO:0033554	Cellular response to stress	2.08	0.0391

**B: Selected GO terms differentially regulated in *w*^1118^ males treated with NIR after 2 h of injury**				

1	GO:0044707	Single-multicellular organism process	1.54	0.0048
2	GO:0022008	Neurogenesis	3.06	0.0071
3	GO:0009987	Cellular process	1.18	0.0088
4	GO:0040011	Locomotion	2.78	0.0127
5	GO:0016043	Cellular component organization	1.56	0.0152
6	GO:0007275	Multicellular organism development	1.53	0.0170
7	GO:0007399	Nervous system development	1.89	0.0173
8	GO:0071840	Cellular component organization or biogenesis	1.50	0.0338

**C: Selected GO terms differentially regulated in *w*^1118^ males treated with NIR after 4 h of injury**				

1	GO:0022008	Neurogenesis	3.07	0.0187
2	GO:0016071	Mrna metabolic process	3.71	0.0204
3	GO:0006725	Cellular aromatic compound metabolic process	1.66	0.0438

*GO terms were sorted based on FDR and ranked accordingly. Tables show selected GO terms changed in females after injury. GOBPID is the ID of the biological process in GO database.*

These data suggest that while NIR exposure could be neuroprotective in brain injury, the effect of this exposure varies by biological sex in *Drosophila*. Male and female flies show differences in gene transcription after injury and such differences are also prevalent after exposure to COX-inhibitory NIR. Overall, both sexes exhibit fewer transcriptional changes in response to TBI after exposure to COX-inhibitory NIR.

### NIR Treatment Downregulates Immune Gene Transcription in Injured Flies

Brain trauma triggers immune system activation, which helps protect tissue against damage (Plesnila, 2016), but long-term inflammation may contribute to neurological deterioration and cognitive decline (Henry et al., 2020). TBI induced neuroinflammation and pathology have also been linked to an increased risk of developing neurodegenerative disorders like AD, PD, and chronic traumatic encephalopathy (CTE) (McKee and Lukens, 2016). We have previously reported an upregulation of immune gene expression in female flies within 4 hr of injury (Shah et al., 2020) and there have been several reports of an upregulated immune response in male flies days, weeks or months after injury (Katzenberger et al., 2016; van Alphen et al., 2018; Swanson et al., 2020). In animal models of TBI, exposure to NIR light has been shown to downregulate pro-inflammatory cytokines and upregulate anti-inflammatory cytokines (Moreira et al., 2009; Khuman et al., 2012; Quirk et al., 2012; Zhang et al., 2014). Based on this evidence, we aimed to explore the effect of modulating mitochondrial COX using NIR exposure at 750 nm on immune gene expression in male and female fly brains inflicted with TBI.

**FIGURE 3:**
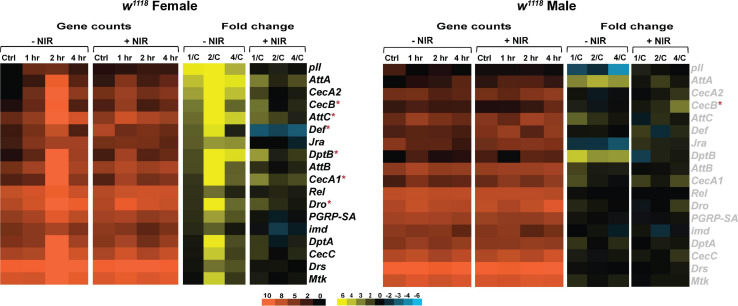
Immune response gene expression is downregulated in injured *w*^1118^ flies with post-NIR exposure. Heatmaps depicting immune response gene expression changes in *w*^1118^ females and males at control, 1, 2, and 4 hr after injury and NIR exposure. The orange scale represents average normalized counts for three replicates in each of the indicated groups. Yellow-blue scale shows fold change for each gene at 1-, 2-, and 4-hr post-injury compared to control. The genes indicated in black font are significantly changed in injured flies not exposed to NIR whereas red asterisk (*) indicates significant alteration in injured flies exposed to NIR (| log2FC| > 1, *p*-value < 0.05). 1/C: Fold change at 1 hr compared to control; 2/C: Fold change at 2 hr compared to control and 4/C: Fold change at 4 hr compared to control. (–NIR: not exposed to NIR and +NIR: exposed to NIR).

The *Drosophila* immune system is regulated by the Toll, Immunodeficiency (Imd) and Janus Kinase protein and the Signal Transducer and Activator of Transcription (JAK-STAT) pathways (West and Silverman, 2017). We looked at transcriptional changes in genes involved with these pathways and observed that *w*^1118^ flies exposed to NIR post-injury exhibit fewer alterations to immune genes than those untreated with NIR (Figure 3). In females, we have previously seen a significant upregulation of genes encoding anti-fungal peptide *Drs* (*Drosomycin)* and anti-bacterial peptides *Diptericin (DptA, DptB), Cecropin (CecA1, CecA2, CecB, and CecC), and Attacin (Att, AttB, and AttC)* (Figure 3A) after injury. *w*^1118^ females exposed to NIR show an upregulation in transcript levels of *CecB*, *AttC*, *DptB*, *CecA1*, and *Dro* in the immediate time frame after injury and are mostly unchanged by 4 hr. The *Drosophila* NF-κB transcription factor *Rel (Relish)* (Hetru and Hoffmann, 2009), a downstream component of the immune deficiency pathway, was significantly upregulated in injured females not exposed to NIR but unchanged after NIR treatment. The NF-κB pathway functions in the host defense of *Drosophila* to control the expression of genes encoding immune-responsive peptides and proteins (Hetru and Hoffmann, 2009). Expression of *Mtk (Metchnikowin)*, an antimicrobial peptide, was seen to be significantly increased in females after TBI but is unchanged in injured females exposed to NIR. Loss of *Mtk* has been shown to reduce mortality and behavioral deficits and improve lifespan in TBI exposed flies (Swanson et al., 2020).

*w*^1118^ males show no transcriptional change in response to injury with or without exposure to NIR for immune response (Figure 3B). We have observed significant upregulation in *CecB* in injured males exposed to NIR 4-hr after injury. Although not significantly induced after injury, we have seen consistently high transcription of *AttA* and *DptB* in injured males not exposed to NIR.

**FIGURE 4:**
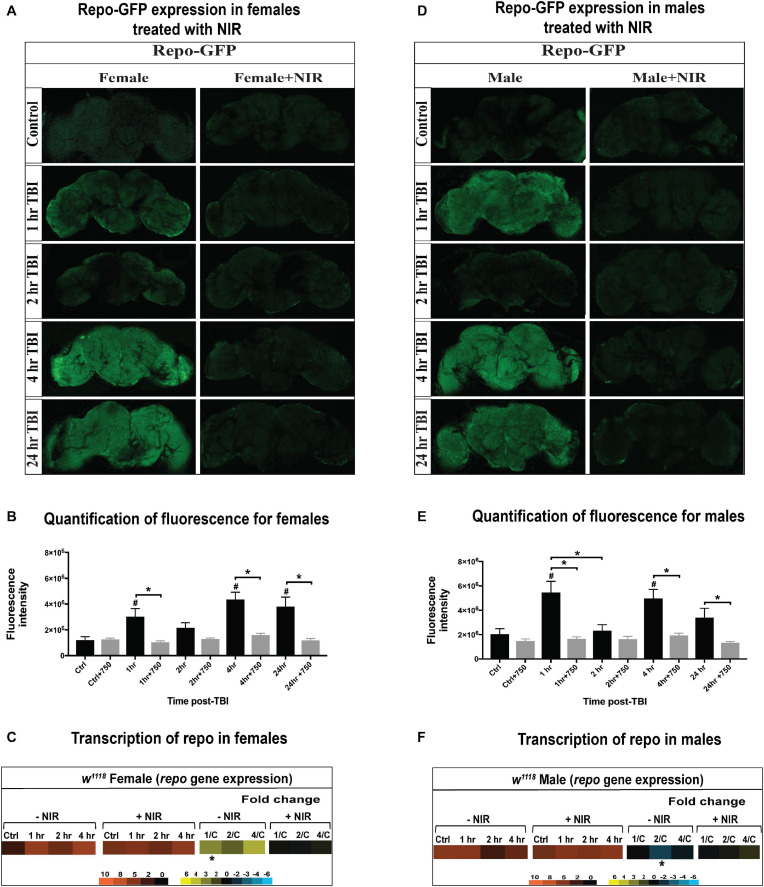
GFP-tagged *repo* expression in *w*^1118^ male and female flies. Confocal images showing change in GFP expression in *Repo*-GFP flies at control, 1, 2, 4, and 24 hr after TBI in females **(A)** and males **(D)** with and without exposure to NIR. About 10–15 brains were imaged for each condition and representative images from each group are shown here. Average fluorescence intensity for each time-point (10–15 brains) was assessed using ImageJ marking whole brain area as region of interest **(B,E)**. A significant increase (One-way ANOVA, Dunnett test, *p*-value < 0.05) in GFP expression was observed for both sexes at 1, 4, and 24 hr after injury. GFP expression was unchanged in both sexes inflicted with TBI after NIR exposure. *Repo* transcription was significantly upregulated (| log2FC| > 1, *p*-value < 0.05) in females 1 hr after injury **(C)** and downregulated in males **(F)** 2 hr after injury. NIR exposure had no change in repo transcription in both sexes.

Trauma-induced changes in glial gene expression is a conserved feature of mammalian (Allen and Barres, 2009; Chung et al., 2015) and *Drosophila* models (van Alphen et al., 2018; Swanson et al., 2020). In flies, glia are able to perform immune related functions and dysregulation of immune signaling in glial cells has been implicated in neurodegeneration (Petersen et al., 2012, 2013). In this study, we utilized the Gal4/UAS system to drive GFP expression in glia using the glial marker *Repo* driving Gal4 and the reporter gene UAS-GFP. Thus, in addition to gene transcription, we looked at change in GFP reporter expression in glial cells after injury and exposure to NIR in both sexes. We have observed differences in GFP expression and repo transcription in both sexes post-TBI (Figure 4). In females (Figures 4A,B) and males (Figures 4D,E), we saw a significant increase in GFP expression 1, 4, and 24 hr after injury but no change at 2 hr compared to control. At all three time-points, the observed increase in GFP expression is significantly decreased after NIR exposure in both sexes. We did observe a significant decrease in GFP expression at 2 hr in males but the regulation of this phasic pattern is not understood. Sex-differences were also observed in *repo* transcription with injured females (Figure 4C) exhibiting significant upregulation 1 hr after injury whereas injured males (Figure 4F) showed significant downregulation 2 hr after injury.

Overall, male and female flies exhibit differences in immune response activation after brain injury with females exhibiting a more immediate alteration in gene transcription than males. Injured males do exhibit an apparent upregulation of immune response as seen by repo-GFP expression, but this response is discordant with repo gene transcription which did not change in males, likely due to changes in protein dynamics (degradation or turnover) not captured by transcriptional data. Inhibiting COX-activity after injury prevents the aberrant activation of immune response gene transcription in females but has no effect on males. It is likely that sexual dimorphism in immune response contributes to the differences in response to injury and NIR exposure.

### Injured Flies Exposed to NIR Exhibit Downregulation in Mitochondrial Gene Transcription

From meeting the increased demand for ATP production to initiating apoptotic signals for clearance of cell debris, mitochondrial function is crucial to the repair process after brain injury (Lifshitz et al., 2004; Hiebert et al., 2015). However, this upregulation in mitochondrial process is also accompanied by an increased generation of ROS, hyperpolarization of ΔΨ_m_ and mitochondrial dysfunction leading to neurodegeneration (Liesa et al., 2009; Shah et al., 2019, 2020). Sexual dimorphism in mitochondrial metabolism is also found to play a crucial role in development of pathologies following injury (Gupte et al., 2019). We have previously shown sex-differences in mitochondrial stress and gene transcription after TBI in *Drosophila* (Shah et al., 2020) and we here wanted to explore the effects of inhibiting mitochondrial COX activity on gene expression in both sexes of injured flies.

**FIGURE 5:**
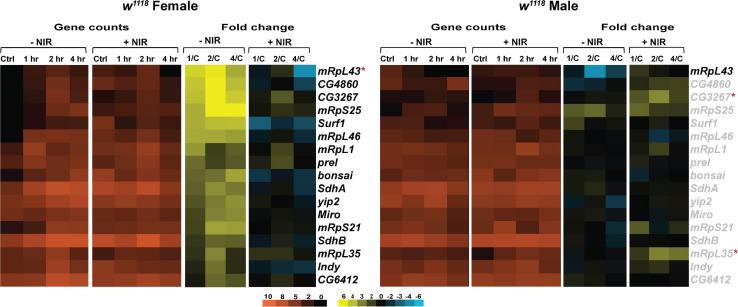
NIR exposure downregulates mitochondrial gene transcription in *w*^1118^ flies subjected to TBI. Heatmaps depicting mitochondrial gene expression changes in *w*^1118^ females and males at control, 1, 2, and 4 hr after injury and NIR exposure. The orange scale represents average normalized counts for three replicates in each of the indicated groups. Yellow-blue scale shows fold change for each gene at 1-, 2-, and 4-hr post-injury compared to control. The genes indicated in black font are significantly changed in injured flies not exposed to NIR whereas red asterisk (*) indicates significant alteration in injured flies exposed to NIR (| log2FC| > 1, *p*-value < 0.05). 1/C: Fold change at 1 hr compared to control; 2/C: Fold change at 2 hr compared to control and 4/C: Fold change at 4 hr compared to control. (–NIR: not exposed to NIR and +NIR: exposed to NIR).

Transcription of genes involved in mitochondrial oxidative phosphorylation, biogenesis, transport and translation is significantly upregulated in injured *w*^1118^ females (Figure 5). We have seen increased expression of *SdhA* and *SdhB* (*Succinate dehydrogenase, subunit A and B*), subunits of the succinate dehydrogenase complex of the ETC after injury in females. *Surf1* (*Surfeit 1*), involved in the assembly of COX is also upregulated in injured females. Increased transcription of ETC genes could indicate upregulation of mitochondrial activity to increase production of ATP (Sen et al., 2017). There is significant upregulation of mitochondrial ribosomal genes like *mRpL43*, *mRpS25*, *mRpL46*, *mRpL1*, *mRpS21*, and *mRpL35* after injury in females. Upregulation in transcription of these genes could indicate alterations in mitochondrial dynamics to clear damage and dysfunctional mitochondria after injury and restore homeostasis. Interestingly, COX inhibition with NIR exposure in females did not have significant transcriptional effects after TBI. There was significant downregulation observed only in *mRpL43* after injury in NIR exposed females. Similar to immune processes, we observed limited changes in injured *w*^1118^ males with or without NIR exposure (Figure 5). *mRpL43* was significantly downregulated in injured males but unchanged after NIR exposure whereas *mRpL35* was significantly upregulated after NIR treatment only. It is possible that in *Drosophila*, similar to humans, mitochondrial function and metabolism vary by sex, which may explain the variations observed between both sexes in response to injury.

These data suggest that modulating mitochondrial COX within the immediate early period following brain injury could prevent mitochondrial damage. However, additional studies that determine ΔΨ_m_, oxygen consumption, ATP production and superoxide production after NIR exposure will be useful to support the observed changes in gene transcription reported here.

### Cytoskeletal Gene Transcription Is Downregulated in Injured Flies After NIR Exposure

Mitochondria move along the axons in both directions using the microtubules and motor proteins (Bartolak-Suki et al., 2017). These interactions between the cytoskeleton and mitochondria are essential for maintaining mitochondrial morphology (Boldogh and Pon, 2006). The twisting and shearing of axons caused by the movement of brain within the skull during an injury is known to cause mechanical deformation on the neuronal cytoskeleton (Hill et al., 2016). Following TBI, Tau, a microtubule associated protein is found to be hyperphosphorylated and impact cytoskeletal integrity and mitochondrial transport (Sivanandam and Thakur, 2012; Edwards et al., 2019). Thus, we wanted to look at the effects of exposure to COX-inhibitory NIR on tau and cytoskeletal gene transcription in injured flies of both sexes.

**FIGURE 6:**
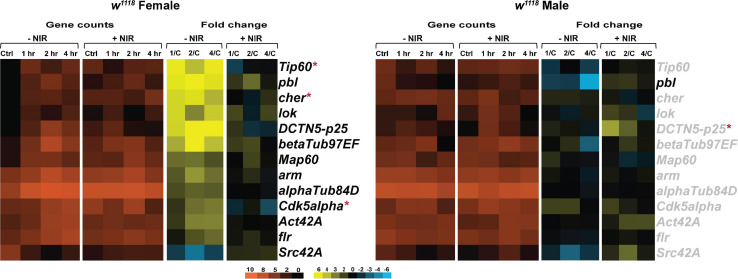
Cytoskeletal gene transcription is downregulated after NIR exposure. Heatmaps depicting cytoskeletal gene expression changes in *w*^1118^ females and males at control, 1, 2, and 4 hr after injury and NIR exposure. The orange scale represents average normalized counts for three replicates in each of the indicated groups. Yellow-blue scale shows fold change for each gene at 1-, 2-, and 4-hr post-injury compared to control. The genes indicated in black font are significantly changed in injured flies not exposed to NIR whereas red asterisk (*) indicates significant alteration in injured flies exposed to NIR (| log2FC| > 1, *p*-value < 0.05). 1/C: Fold change at 1 hr compared to control; 2/C: Fold change at 2 hr compared to control and 4/C: Fold change at 4 hr compared to control. (–NIR: not exposed to NIR and +NIR: exposed to NIR).

As observed in immune response and mitochondrial gene transcription, we saw significant upregulation in expression of cytoskeletal genes in injured females (Figure 6). *w*^1118^ injured females exhibit significant upregulation of kinases involved in Tau phosphorylation like *lok* (*loki*) and *Cdk5alpha* (*Cdk5 activator-like protein*) after injury (Figure 6). Cyclin-dependent kinases were shown to be involved in mitophagy in cell culture models (Moskal et al., 2020). Microtubule integrity depends largely on tubulin polymerization (Bartolak-Suki et al., 2017) and we found a significant upregulation in *alphaTub84D* (α*-Tubulin at 84D*) and *betaTub97EF* (β*-Tubulin at 97EF*), both involved in polymerization of microtubules. In *w*^1118^ injured males (Figure 6), we observed very little transcriptional change with only *pbl* (*pebble*), involved in actin cytoskeleton reorganization being significantly downregulated whereas *pbl* was upregulated in injured females. Exposure to NIR resulted in significant alteration in *Tip60* (*Tat interactive protein 60kDa*), *cher* (*cheerio*) and *Cdk5alpha* in females. *DCTN5-p25 (Dynactin 5, p25 subunit*), a motor protein involved in retrograde axonal transport was significantly upregulated in injured females but unchanged after NIR exposure. *DCTN5-p25* was significantly upregulated after NIR exposure in injured males.

Our data suggests that brain injury affects cytoskeletal integrity differently in male and female flies and NIR exposure limits the damage sustained by trauma in both sexes.

## Discussion

Pharmacological therapies for brain injury are primarily focused on modulating major neurotransmitter processes to enhance the neurocognitive sequelae of TBI (Morries et al., 2015). Unfortunately, little has been found to reverse TBI damage caused by mitochondrial dysfunction, a well-known cause of neuronal death (Hiebert et al., 2015; Morries et al., 2015). In this study we employed NIR light, a non-pharmacological approach to minimize mitochondrial damage after TBI in *Drosophila*. NIR treatment has been shown to provide benefit in animal models of ischemia/reperfusion injury (Sanderson et al., 2018), spinal cord injury (Giacci et al., 2014), stroke (Strubakos et al., 2020), optic nerve injury (Giacci et al., 2014), and in human trials of TBI (Naeser et al., 2014). Sex differences in outcome to TBI have been observed in flies (Shah et al., 2020) and there exist strong evidence indicating sex differences in mitochondria could also have an effect on TBI response (Conley et al., 2014; Gupte et al., 2019). Hence, we assessed the effects of modulating mitochondrial COX using NIR exposure on gene transcription in the immediate time frame after brain injury in both sexes. The fly brain is enclosed in a cuticle composed of chitin which strongly absorbs the 750 nm infrared radiation (Klocke et al., 2011), so we sequenced 3′mRNA libraries of isolated *w*^1118^ fly brains at control, 1, 2, and 4 hr after TBI. Our results suggest that inhibiting COX activity downregulates gene expression in injured females whereas injured males exhibit minimal changes with or without NIR treatment.

Different studies have suggested a positive effect of PBM treatment in TBI patients (Naeser et al., 2014; Poiani et al., 2018; Carneiro et al., 2019). NIR between 700–1000 nm readily penetrates the scalp and skull and has the potential to improve cellular activity of compromised brain tissue (Santos et al., 2018). Most studies employing NIR treatment have focused on evaluating the efficacy of this exposure on neuropsychological defects like impaired cognition and mood arising from brain injury (Poiani et al., 2018; Santos et al., 2018) but none have looked at the effect of NIR on gene transcription. Since failing mitochondria can place the cell in energy crisis, mitochondrial dysfunction after injury could affect gene transcription, an energy demanding process (Muir et al., 2016). Thus, we looked at gene transcription profile to identify the effect of mitochondrial damage on brain transcriptome post-TBI and COX inhibition. It should be noted that most PBM studies conducted to date used COX-activating NIR, such as 810 nm, and no COX-inhibitory wavelengths were known until their recent discovery by the Hüttemann laboratory (Sanderson et al., 2018). Therefore, the goal of this study was to assess (a) the change in gene expression in response to COX-inhibitory NIR exposure and (b) whether the response varies in both sexes. Contrary to other studies, we have made use of a single COX-inhibitory wavelength (750 nm) to assess genome-wide changes in gene expression in TBI inflicted fly brains. We specifically looked at alteration in genes involved in immune response, mitochondrial function and cytoskeleton after TBI.

Brain injury causes a prolonged activation of inflammatory responses, which exacerbates primary damage occurring within hours of injury (McKee and Lukens, 2016; Plesnila, 2016; Dinet et al., 2019). Efforts to manipulate genes or pathways that can either trigger anti-inflammatory responses or inhibit pro-inflammatory processes have been underway as means to curb this extensive brain damage (Griffin, 2011; McKee and Lukens, 2016). A growing body of evidence also links mitochondrial dysfunction with increased incidences of immune activation (Walker et al., 2014; Breda et al., 2019). Here, we see an increased expression of genes involved in the *Drosophila* immune system after injury in females and most of these upregulated genes are unchanged after NIR exposure. Previous studies have also noted protective effects of PBM on immune system in TBI models (Moreira et al., 2009; Hennessy and Hamblin, 2017) but the underlying cause for differences between males and females as observed here remains unknown. *Drosophila* males have been shown to have increased immune gene transcription days or weeks after injury (Katzenberger et al., 2016; Swanson et al., 2020), so it is likely that an upregulated gene expression could be observed if longer time points are assessed. Sexual dimorphism in mitochondria (Demarest and McCarthy, 2015; Gupte et al., 2019) could also be influencing the secondary damage cascades after brain injury but further studies are required to explore such differences in flies. It should also be noted that the NIR wavelength (COX-inhibiting) and energy density employed in this study is different to previously published reports (Weinrich et al., 2017), which could also have an impact on the response. Weinrich et al. (2017) showed that lifelong 670-nm exposure in *Drosophila* extends lifespan and improves aged mobility. However, our initial assessments determined that chitin absorbed NIR at the 750-nm exposure, thus raising the possibility that different wavelengths have varying penetration through tissues. Due to this we were unable to assess behavioral function or survival post-TBI in NIR-exposed flies, however our focus was to assess gene expression changes in the brain after injury.

One of the most pronounced effect of TBI is axonal damage which impacts the cytoskeletal integrity and structure (Fitzpatrick et al., 1998; Saatman and Burkhardt, 2009). The cytoskeleton regulates mitochondrial positioning and transport and this interaction of mitochondria with microtubules is a tightly regulated process (Anesti and Scorrano, 2006; Boldogh and Pon, 2006). An additional level of regulation is accomplished by microtubule-associated proteins (MAP’s) like Tau (Ebneth et al., 1998) and pathological Tau, a common finding in TBI patients and animals models of brain injury (Collins-Praino and Corrigan, 2017; Castellani and Perry, 2019), inhibits mitochondrial transport (Shahpasand et al., 2012). In this study, we observed an upregulation in several cytoskeletal genes in injured females but no change in tau transcription in both sexes after TBI. NIR exposure appears to show an immediate response in females but minimal change in males. Similar trend in gene expression is also observed in mitochondrial gene expression for both sexes after injury and subsequent NIR exposure. Since tau transcription was unaffected by brain injury, we also exposed TBI inflicted Tau knockout flies to NIR and observed mostly downregulation or no change in gene expression in both sexes (Supplementary Data 1). The synergistic effect of absence of tau and NIR exposure in injured flies highlights the role of tau expression and mitochondrial dysfunction in TBI outcome.

In conclusion, we have shown that exposure to COX-inhibitory NIR light within the immediate timeframe after brain injury downregulates gene expression in injured flies. The response to TBI and the subsequent modulation of mitochondrial COX by exposure to NIR varies by biological sex with females exhibiting a more pronounced effect than males in *Drosophila*. Although the cause of these transcriptional differences remains unknown, the presence of metabolic tissues, sex-specific genes or brain architecture could be potential factors requiring further studies. In this study, we were unable to assess lifespan or behavioral measures in NIR-exposed flies due to the absorbance property of chitin, but we present genome-wide transcriptional changes in *Drosophila* brains to decipher gene networks or pathways that are affected by TBI and NIR. Finally, we propose that a detailed understanding of the disease mechanism at the mitochondrial level after acute stress (Sanderson et al., 2013, 2018) will make possible use of COX-inhibitory and -activating NIR at the early and late time periods following tissue injury, respectively, to limit ΔΨ_m_ hyperpolarization and thus ROS early to suppress cell death, whereas at later chronic stages of the disease, COX-activating NIR may be useful to enhance tissue remodeling and repair.

## Data Availability Statement

The datasets presented in this study can be found in online repositories. The names of the repository/repositories and accession number(s) can be found in the article/Supplementary Material. Gene expression data are available in the GEO database under accession number GSE140663 (*w*_*1118*_ without NIR exposure) and GSE158061 (*w*_*1118*_ with NIR exposure).

## Author Contributions

DR, MH, and KG conceived the project. ES and KG conceptualized the content. ES performed the data analysis and wrote the manuscript. All authors edited the manuscript and assisted with data analysis.

## Conflict of Interest

MH and TS are co-founders of Mitovation Inc., that develops infrared light therapy for ischemia/reperfusion injury applications. The remaining authors declare that the research was conducted in the absence of any commercial or financial relationships that could be construed as a potential conflict of interest.

## Supplementary Material

Supplementary Data Sheet 1 Gene Ontology tables.
